# When Drainage is Not Enough in Emphysematous Pyelonephritis Complicated by Necrotizing Fasciitis: A Case Report

**DOI:** 10.7759/cureus.104189

**Published:** 2026-02-24

**Authors:** Jerry H Rose, Julian A Secondino, Carlos M Rey

**Affiliations:** 1 Clinical Medicine, American University of Antigua, Osbourn, ATG; 2 Radiology, Hialeah Hospital, Miami, USA; 3 Psychiatry, American University of Antigua, Osbourn, ATG

**Keywords:** emphysematous pyelonephritis (epn), laboratory risk indicator for necrotizing fasciitis (lrinec) score, necrotizing fasciitis management, necrotizing fasciitis with toxic shock syndrome, percutaneous nephrostomy tube

## Abstract

Necrotizing fasciitis (NF) is a rapidly progressive, life-threatening soft tissue infection characterized by widespread fascial necrosis, systemic toxicity, and high mortality without prompt surgical intervention. Emphysematous pyelonephritis (EPN) is a rare necrotizing infection of the kidney and perirenal tissues caused by gas-producing microbes, most often *Escherichia coli*, in patients with uncontrolled diabetes mellitus. We report the case of a 69-year-old man who developed NF with contiguous EPN. Despite aggressive surgical and medical management, he rapidly progressed to refractory septic shock and multi-organ failure, ultimately resulting in death. This case highlights the importance of early recognition of NF and determining its underlying cause, as delays in intervention may lead to uncontrollable sepsis.

## Introduction

Necrotizing fasciitis (NF) is an aggressive soft tissue infection that spreads along fascial planes. Even with prompt treatment, NF carries a mortality of 29-70% [1]. NF is usually seen in the setting of trauma in elderly patients with underlying diabetes or immunocompromised status. Incidence is 3-5 cases per 100,000 in developed countries [1]. Diagnosis requires a high index of suspicion, as initial symptoms may mimic those of cellulitis or a simple soft tissue infection. Subcutaneous emphysema, rapid pain escalation, pain out of proportion to exam, and systemic toxicity are key diagnostic clues to this underlying disease process [1].

Emphysematous pyelonephritis (EPN) is a severe, necrotizing infection of the renal parenchyma and perirenal tissues. It is most often caused by *E. coli* in diabetic patients and carries a mortality of 20-40% despite antibiotics and surgical intervention [2-5]. Rarely, EPN may extend beyond the retroperitoneum, giving rise to necrotizing fasciitis [2,4,5].

In this case report, we describe a patient with EPN complicated by rapidly progressive NF, illustrating the therapeutic challenges that arise when EPN extends beyond the retroperitoneum into the thigh. NF was the presenting feature of EPN here, though this combined occurrence is rare, with only thirteen cases reported in the literature [6-18]. Despite urgent surgical debridement, percutaneous nephrostomy, and fasciotomy, the patient failed to stabilize and remained dependent on mechanical ventilation and vasopressor support until death.

## Case presentation

A 69-year-old male with known Type II diabetes and hypertension presented to the emergency department for left thigh pain and swelling associated with lower back pain. The patient was obtunded and unable to provide a reliable medical history at admission. His symptoms began three months ago following a mechanical fall and have gradually worsened. The patient lost his balance while climbing stairs, fell into a seated position, and injured his leg. He denied any loss of consciousness. Prior to this presentation, the patient saw an orthopedic surgeon and received an analgesic injection, but his leg pain progressively worsened.

On arrival to the emergency department, his vital signs were: temperature 97.9 °F, blood pressure 78/47 mmHg, heart rate 125 beats per minute, respiratory rate 22 per minute, and SpO_2_ 86% on 6 L/minute via nasal cannula. He reported severe leg pain. He was oriented to time and person but not place. Physical exam revealed gangrenous changes involving the left medial and posterior thigh. The overlying skin demonstrated large areas of black eschar interspersed with dusky, violaceous discoloration. The surrounding tissue showed epidermal sloughing and peeling, with patches of necrotic, malodorous tissue. The margins were irregular, with progression beyond clearly demarcated borders, consistent with rapidly advancing necrotizing infection. Palpation elicited diffuse tenderness from the left hip down to the left leg, with surrounding erythema and induration extending proximally toward the groin. The range of motion of the left hip and leg was decreased due to pain. Admission and postoperative laboratory values, along with key interventions, are summarized in Tables 1, 2, 3.

**Table 1:** Clinical Timeline With Key Laboratory Values. Patients received intravenous fluid resuscitation in the emergency department. Reference ranges reflect institutional laboratory standards.

Parameters	Values	Reference Range
White blood cell count (WBC)	22.7 ×10³/µL	4.0-11.2 ×10³/µL
Hemoglobin (Hgb)	8.3 g/dL	13.7-17.5 g/dL
Creatinine (Cr)	2.8 mg/dL	0.7-1.3 mg/dL
Lactate	2.9 mmol/L	0.7-2.0 mmol/L
C-reactive protein (CRP)	236.2 mg/L	<10 mg/L

**Table 2:** Operating Room Course. The patient underwent surgical debridement and percutaneous nephrostomy, required mechanical ventilation and vasopressor support, and was initiated on broad-spectrum empiric antimicrobial therapy.

Parameters	Values	Reference Range
Surgical procedures	Thigh debridement; percutaneous nephrostomy	-
Airway management	Mechanical ventilation	-
Hemodynamic support	Norepinephrine infusion	-
Empiric antibiotics	Cefepime 2 g IV; Metronidazole 500 mg IV; Linezolid 600 mg IV	Standard dosing

**Table 3:** Intensive Care Unit Laboratory Values (Postoperative Day One). Postoperatively, the patient required ventilatory and vasopressor support. Antimicrobial therapy was escalated to clindamycin 600 mg IV every 8 hours, vancomycin 1 g IV every 12 hours, and ceftriaxone 1 g IV every 24 hours. Reference ranges reflect institutional laboratory standards.

Parameters	Values	Reference Range
White blood cell count (WBC)	37.2 ×10³/µL	4.0-11.2 ×10³/µL
Hemoglobin (Hgb)	6.6 g/dL	13.7-17.5 g/dL
Creatinine (Cr)	2.4 mg/dL	0.7-1.3 mg/dL
Sodium	137 mEq/L	135-145 mEq/L
Glucose	197 mg/dL	70-99 mg/dL
Lactate	4.3 mmol/L	0.7-2.0 mmol/L
Blood culture/Gram stain	Gram-negative rods (suggestive of Escherichia coli)	Negative

**Figure 1:**
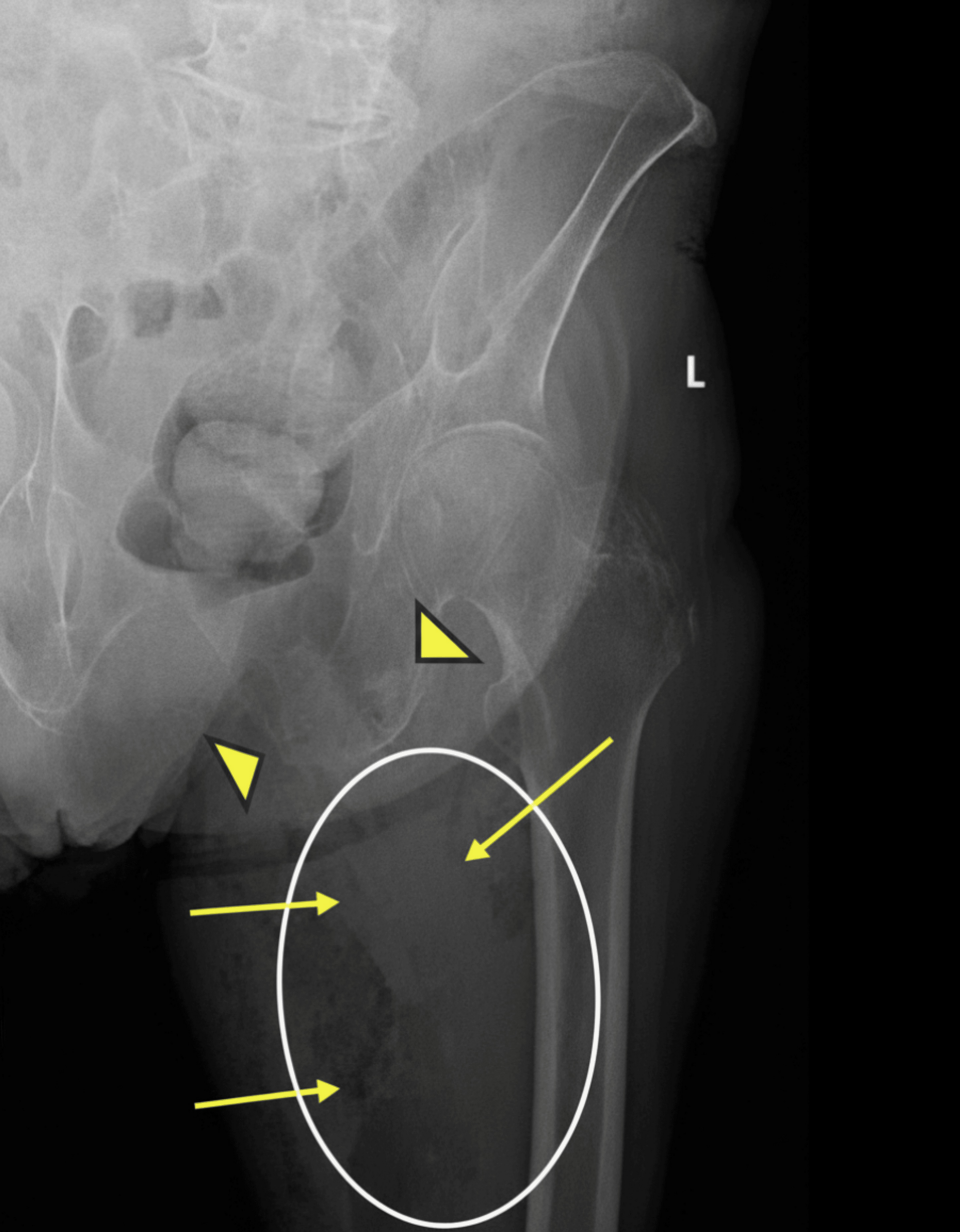
Left Femur X-ray AP View. Gas within the left thigh (yellow arrows, white oval). Arrowheads point to a healed fracture of the left inferior ramus.

STAT left thigh X-ray revealed soft tissue edema and gas extending within the left psoas muscle and thigh with evidence of abscess formation (Figure 1).

**Figure 2:**
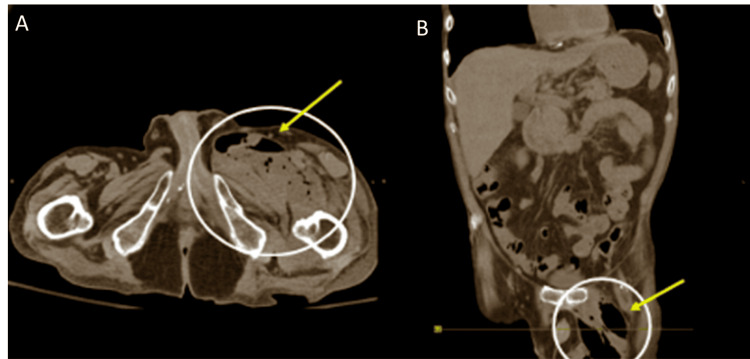
Axial and Coronal Non-contrast CT Images of the Abdomen and Pelvis. (A, B) Soft tissue gas with edema tracking along the left iliopsoas fascial plane and intermuscular septa. White circles outline soft tissue thickening in the left thigh. Yellow arrows point to an extensive circumferential gas collection within the adductor muscles.

Non-contrast CT abdomen and pelvis corroborated the finding of gas and edema tracking along the iliopsoas (Figure 2).

**Figure 3:**
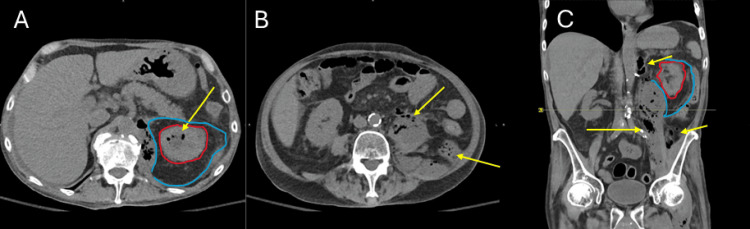
Non-contrast CT of the Abdomen and Pelvis Demonstrating Emphysematous Pyelonephritis. (A, B) Axial images showing mottled gas within the ureters and pelvicalyceal system with extension into the retroperitoneum, involving the superior aspect of Gerota’s fascia and the iliopsoas muscle. (C) Coronal image showing extensive fluid collection in the left perirenal space is outlined in blue. Diffuse renal parenchymal congestion is outlined in red. Yellow arrows indicate areas of gas formation.

Non-contrast CT of the abdomen and pelvis revealed gas in the edematous renal parenchyma, collecting system, and retroperitoneum, with tracking into the iliopsoas fascia (Figure 3).

Contrast-enhanced imaging was deferred in the setting of acute kidney injury and hemodynamic instability, as administration of contrast under these conditions carries a risk of further renal deterioration and clinical decompensation. Image findings were consistent with necrotizing fasciitis and EPN.

The patient was rushed to the OR in septic shock and started on broad-spectrum intravenous antibiotics, aggressive fluid resuscitation with Ringer’s Lactate and KCl, and norepinephrine infusion. Emergent surgical debridement of necrotic tissue in the left groin and thigh was performed, where the wound was cleansed, irrigated, and left open to drain, with clean margins to remove all areas of necrotizing tissue. A percutaneous nephrostomy tube was placed to drain the perirenal fluid collection.

Postoperatively, the patient was transferred to the ICU and remained intubated and mechanically ventilated for pressure support. On postoperative day one (POD1), the patient’s laboratory markers for sepsis worsened despite infectious disease Society of America guideline-directed medical and surgical management [19]. Blood cultures isolated *E. coli*, so the antibiotics were narrowed accordingly. However, by POD3, he developed acute respiratory distress syndrome, refractory septic shock on two vasopressors, worsening metabolic acidosis, severe hypoglycemia, anemia, thrombocytopenia, and multiorgan failure. One week after admission, on POD6, he remained on a ventilator with a blood pressure of 40/30 mmHg and was very lethargic and subsequently expired.

## Discussion

The coexistence of EPN and NF is extremely rare and carries an abysmal prognosis. Our patient presented with radiographic and intraoperative findings consistent with both conditions, which likely compounded the rapid progression to septic shock and multi-organ failure.

Diagnostic considerations

The Laboratory Risk Indicator for Necrotizing Fasciitis (LRINEC) is a tool used to distinguish NF from other soft tissue infections. The score assigns a value of 0-2 to six laboratory parameters: C-reactive protein, hemoglobin, leukocyte count, serum sodium, serum creatinine, and blood glucose level. Although the LRINEC score should not be used in isolation, higher scores are associated with a greater likelihood of necrotizing fasciitis in the appropriate clinical context. A score greater than 6 indicates a high probability of NF [20].

In our patient, the LRINEC score was 10, placing him in the high-risk category, concordant with his rapid clinical deterioration, imaging findings, and operative confirmation of necrotizing fasciitis [21]. Polymicrobial infection is the most common cause of NF, frequently involving gram-negative and anaerobic organisms, with *Streptococcus pyogenes, Clostridium perfringes, Vibrio vulnificus,* and *Escherichia Coli* as common culprits. The isolation of *Escherichia coli* in our patient’s blood cultures is consistent with this pattern and aligns with prior reports demonstrating an advanced disease course at the time of diagnosis [22].

Despite transfusion and vasopressor support, the patient demonstrated persistent hemodynamic instability and worsening laboratory anomalies. These findings suggest sepsis-related complications: capillary leak syndrome and intravascular hemolysis. Whereby, circulating bacterial toxins cause endothelial damage and loss of circulating albumin, and hemolysins reduce hematocrit in the absence of an identifiable source of bleeding [1]. Treatment failure should prompt reevaluation.

Treatment strategies

The patient underwent appropriate initial management with antibiotics, surgical debridement of the thigh, and percutaneous nephrostomy drainage. However, he remained in refractory septic shock despite these measures. Current evidence suggests that in such circumstances, nephrectomy should be considered as definitive source control [21-23]. A risk-benefit analysis is presented in Table 2. Timely nephrectomy improves survival despite considerable surgical risk [22,24,25].

**Table 4:** Risk–benefit Analysis of Nephrectomy in EPN. [21-23,26-28]

Nephrectomy	Details
Indications	Huang–Tseng class 3b/4 (perirenal or extra-renal gas)
	Persistent septic shock despite antibiotics + drainage
	Poor prognostic factors: renal dysfunction, thrombocytopenia, altered mentation, rising lactate
	Treatment failure with conservative management
Risks	Perioperative mortality in an unstable patient
	Loss of renal mass → risk of dialysis
	Hemorrhage, wound complications, anesthetic risk
	Post-operative complications, prolonged ICU stay
Benefits	Treatment failure with conservative management
	Definitive source control of necrotizing renal infection
	Reduced mortality in refractory EPN after failed drainage [6–8,10–12]
	Interrupts bacteremia and endotoxemia, driving shock
	Prevents contiguous spread to fascia (secondary NF)
Perioperative Mitigation Strategies	Hemodynamic optimization with vasopressors, fluid resuscitation
	Early dialysis access if AKI/severe acidosis present
	Broad-spectrum antibiotics pre and post-operatively, de-escalate by culture
	Multidisciplinary coordination (urology, acute care surgery, ICU, anesthesia)

Poor prognostic variables indicate a need to escalate therapy. In our patient, these included advanced EPN (Huang-Tseng class 3b or 4), presence of comorbid diabetes, renal dysfunction, altered mental status, thrombocytopenia, and persistent sepsis despite percutaneous drainage [26-28]. Worsening laboratory values (metabolic acidosis, worsening lactate, progressive leukocytosis, anemia, and thrombocytopenia) warranted consideration of rescue therapy. Although nephrectomy carries a very high morbidity and mortality risk, surgical escalation may have been beneficial in this case, as the potential mortality benefit outweighed the severe morbidity of emergency nephrectomy in critically ill patients.

This case underscores several important teaching points. First, clinicians must recognize that although percutaneous drainage is often adequate for localized EPN, the presence of systemic deterioration should prompt consideration of nephrectomy without delay. Second, the extension of EPN into adjacent fascial planes is a rare but devastating complication that mandates multidisciplinary coordination between urology, surgery, and critical care teams. Finally, timely escalation of care, moving from guideline-based conservative therapy to more aggressive surgical intervention, could be lifesaving in otherwise catastrophic infections.

## Conclusions

NF and EPN are both rare but life-threatening conditions. Their coexistence significantly worsens prognosis. One should be aware that NF can be a presenting feature of EPN. While antibiotics, percutaneous drainage, and debridement are essential first steps, refractory sepsis is associated with high mortality, and nephrectomy may be necessary in advanced EPN. This case highlights the difficulties in managing EPN, complicated by NF.
